# Voruciclib, a clinical stage oral CDK9 inhibitor, represses MCL-1 and sensitizes high-risk Diffuse Large B-cell Lymphoma to BCL2 inhibition

**DOI:** 10.1038/s41598-017-18368-w

**Published:** 2017-12-21

**Authors:** Joyoti Dey, Thomas L. Deckwerth, William S. Kerwin, Joseph R. Casalini, Angela J. Merrell, Marc O. Grenley, Connor Burns, Sally H. Ditzler, Chantel P. Dixon, Emily Beirne, Kate C. Gillespie, Edward F. Kleinman, Richard A. Klinghoffer

**Affiliations:** 1 Presage Biosciences, Inc, Seattle, WA USA; 2 Edward F. Kleinman LLC, Pawcatuck, CT USA

## Abstract

Aberrant regulation of BCL-2 family members enables evasion of apoptosis and tumor resistance to chemotherapy. BCL-2 and functionally redundant counterpart, MCL-1, are frequently over-expressed in high-risk diffuse large B-cell lymphoma (DLBCL). While clinical inhibition of BCL-2 has been achieved with the BH3 mimetic venetoclax, anti-tumor efficacy is limited by compensatory induction of MCL-1. Voruciclib, an orally bioavailable clinical stage CDK-selective inhibitor, potently blocks CDK9, the transcriptional regulator of MCL-1. Here, we demonstrate that voruciclib represses MCL-1 protein expression in preclinical models of DLBCL. When combined with venetoclax *in vivo*, voruciclib leads to model-dependent tumor cell apoptosis and tumor growth inhibition. Strongest responses were observed in two models representing high-risk activated B-cell (ABC) DLBCL, while no response was observed in a third ABC model, and intermediate responses were observed in two models of germinal center B-cell like (GCB) DLBCL. Given the range of responses, we show that CIVO, a multiplexed tumor micro-dosing technology, represents a viable functional precision medicine approach for differentiating responders from non-responders to BCL-2/MCL-1 targeted therapy. These findings suggest that the combination of voruciclib and venetoclax holds promise as a novel, exclusively oral combination therapy for a subset of high-risk DLBCL patients.

## Introduction

Diffuse large B-cell lymphoma (DLBCL), one of the most common forms of non-Hodgkin lymphoma, is generally responsive to the R-CHOP regimen (rituximab combined with cyclophosphamide, doxorubicin, vincristine and prednisone)^1^. However, approximately 40% of patients continue to exhibit chemo-refractory DLBCL or relapse and ultimately succumb to their disease^1,2^. Differences in tumor cell-of-origin and underlying genetic drivers contribute to variabilities observed in patient responses to R-CHOP^3,4^. The three most well characterized molecular subtypes of DLBCL include germinal center B-cell like (GCB), primary mediastinal B cell lymphoma (PMBL) and activated B-cell (ABC) subtypes^4^. The ABC-subtype, the most aggressive form of DLBCL, tends to be the least responsive to R-CHOP based therapy and represents a clear unmet need^3,5,6^.

Lack of response in DLBCL is at least in part due to evasion of apoptosis, a hallmark of cancer, often resulting from aberrations in pro-survival BH3 proteins of the B-cell leukemia/lymphoma-2 (BCL-2) family^7,8^. The BCL-2 family consists of structurally related regulators of programmed cell death, of which those with functionally redundant anti-apoptotic activity include BCL-2 itself, BCL-xL, and MCL-1^9^. ABC subtype tumors have significantly higher BCL-2 expression compared to the GCB subtype and high BCL-2 expression is considered a poor prognostic indicator^5,10^. Therapeutically, selective inhibition of BCL-2 has been achieved with the BH3 mimetic, venetoclax (ABT-199), resulting in high response rates in certain lymphoid malignancies including chronic lymphoid leukemia (CLL) and mantle cell lymphoma (MCL)^11,12^. However, exclusive targeting of BCL-2 with venetoclax in relapsed or refractory non-Hodgkin lymphoma (NHL) patients has yielded only a modest 18% overall response rate with a 1-month median progression-free survival^11^. One primary mechanism of resistance to venetoclax is overexpression of MCL-1 which, by virtue of functional redundancy, compensates for loss of BCL-2 activity^13^.

One of the most commonly amplified genes in human cancer, MCL-1, has been shown to be either amplified at the genomic level or over-expressed in a significant fraction of DLBCL patients, making it an important candidate for therapeutic targeting particularly in patients who fail on R-CHOP^8,14^. Furthermore, chemotherapy or chronic BCL-2 inhibition in tumors initially sensitive to venetoclax may also lead to compensatory upregulation of MCL-1 and consequent emergence of drug-refractory cancer cells^15^. Therefore, combined targeting of BCL-2 and MCL-1 is a potential treatment strategy especially in aggressive lymphomas that are refractory to standard chemotherapy^16–18^.

Inhibition of CDK9 constitutes a promising approach for inhibiting MCL-1 function. CDK9 is a key component of the positive transcriptional regulator complex pTEFb which activates transcription via phosphorylation of RNA polymerase II (RNA POL II)^19^. Inhibition of CDK9 blocks transcription resulting in the repression of short-lived proteins such as MCL-1^19^. While direct inhibition of MCL-1 is feasible, existing MCL-1 inhibitor programs are still in the early stages of development^20–25^. Therefore, depletion of MCL-1 by CDK9 inhibitors may currently represent the most clinically advanced means to inactivate this pro-survival BH3 family member.

Here we demonstrate that the small molecule flavone derivative, voruciclib, a clinical stage CDK inhibitor, targets CDK9 with sub-nanomolar biochemical potency and represses expression of MCL-1 in multiple models of DLBCL. The safety profile and tolerability of voruciclib has been established by two independent Phase 1 clinical trials^26,27^. Furthermore, voruciclib is distinguished from other CDK9 inhibitors such as flavopiridol or dinaciclib by its oral bioavailability, making it an exceptionally attractive candidate for clinical development. This positions voruciclib favorably for combination therapies in malignancies that are reliant on MCL-1. Consistent with this strategy, we demonstrate that voruciclib combines with venetoclax to induce synergistic tumor cell death and durable tumor remission in preclinical models of DLBCL, including the high-risk ABC subtype.

## Results

### Voruciclib exhibits a more selective target profile than flavopiridol with sub nanomolar potency against CDK9

**Figure 1:**
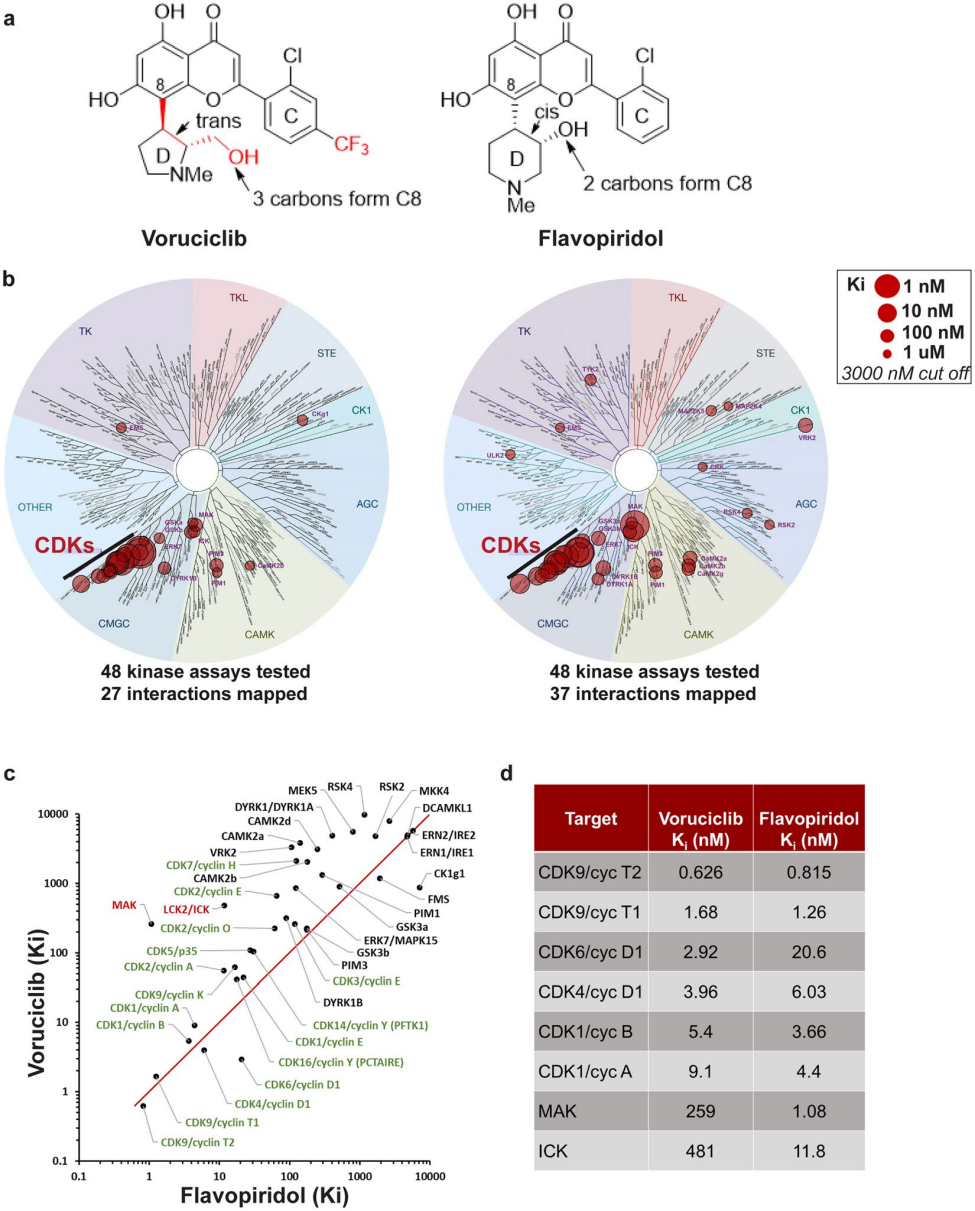
Comparative kinase profiles of voruciclib and flavopiridol. (**a**) Notable differences in the structures of voruciclib and flavopiridol - First, in the C ring, voruciclib contains a trifluoromethyl (CF3) group at the 4-position of the phenyl ring (shown in red). Second, the D rings of the two molecules contain different saturated heterocycles, each with a basic nitrogen and a hydroxyl or hydroxymethyl substituent: 2-hydroxymethyl-1-methylpyrrolidin-3-yl (voruciclib) and 3-hydroxy-1-methylpiperidiny-4-yl (flavopiridol). Third, the hydroxyl group of voruciclib is connected to C8 by 3 carbons whereas that of flavopiridol is connected by 2 carbons. **(b)** Kinase dendograms for voruciclib and flavopiridol - Of the 48 consensus hits from the DiscoverRx and Thermofisher kinase screens that were followed up for functional IC50 determination (Reaction Biology), the kinases which bound at K_i_ values under 3000 nM for voruciclib and flavopiridol respectively are shown in the dendograms with red circles, where larger circles indicate higher-affinity binding. **(c,d)** Comparison of K_i_ values for voruciclib and flavopiridol targets shows CDK targets (green) and non-CDK targets (black). High affinity non-CDK targets of flavopiridol (ICK and MAK) are shown in red.

Voruciclib is structurally similar to the flavonoid-based CDK inhibitor flavopiridol, but differences in the substitution (CF3) and structure (hydroxypiperidine vs. hydroxymethyl pyrrolidine) of the C and D rings, respectively, of the flavonoid scaffold may differentiate the target profiles of these two molecules (Fig. 1a). The largest potential for voruciclib and flavopiridol to exhibit differential inhibitory profiles with respect to other CDKs and kinases likely rests with the D ring. The D ring of flavopiridol has previously been shown to form highly defined interactions in the phosphate binding pocket, based on the X-ray structure of a flavopiridol analog bound to CDK2^28^. Differences in ring size, substitution, stereochemistry, conformation and disposition of the hydroxyl (from C8) between voruciclib and flavopiridol are likely to alter binding to the active site of CDKs and other kinases. This raises the possibility that voruciclib may have attenuated inhibitory activity against certain kinases which are potently inhibited by flavopiridol and vice versa.

To characterize the target profile of voruciclib we first performed two independent kinase assay screens (DiscoveRx ScanMax and Thermofisher SelectScreen, 468 and 414 kinases respectively). Both screens were performed at high (10 μM) and low (50 nM) concentrations of voruciclib. To prioritize the kinases for follow-up in a functional IC_50_ screen, IC_50_ values were estimated for each of the two inhibitor concentrations using a Hill coefficient of 1 and the estimates from both screens were compared. This yielded 48 consensus hits for which IC_50_ values for both voruciclib and flavopiridol were then established by performing 10-point dose-response curves (Reaction Biology). K_i_ values for each target were calculated and compared for the two drugs (Fig. 1b–d). As expected, both molecules demonstrate selectivity for the CDK subfamily of kinases with most potent activity against CDKs 9, 6, 4, and 1. However, consistent with the hypothesis that structural differences of the D ring may alter target selectivity, voruciclib exhibits significantly reduced activity against kinases outside of the CDK family compared to flavopiridol (Fig. 1b,c). Particularly notable is the substantially low activity of voruciclib against two highly related kinases, ICK and MAK, when compared to flavopiridol. While flavopiridol exhibits equipotent activity against MAK and favored target CDK9, voruciclib exhibits 100-fold greater selectivity for CDK9 than MAK (Fig. 1c). Thus, voruciclib is a CDK-selective inhibitor with potent activity against CDK9 and less non-CDK off-target liability than flavopiridol.

### Voruciclib inhibits CDK9 target, MCL-1 in DLBCL

**Figure 2:**
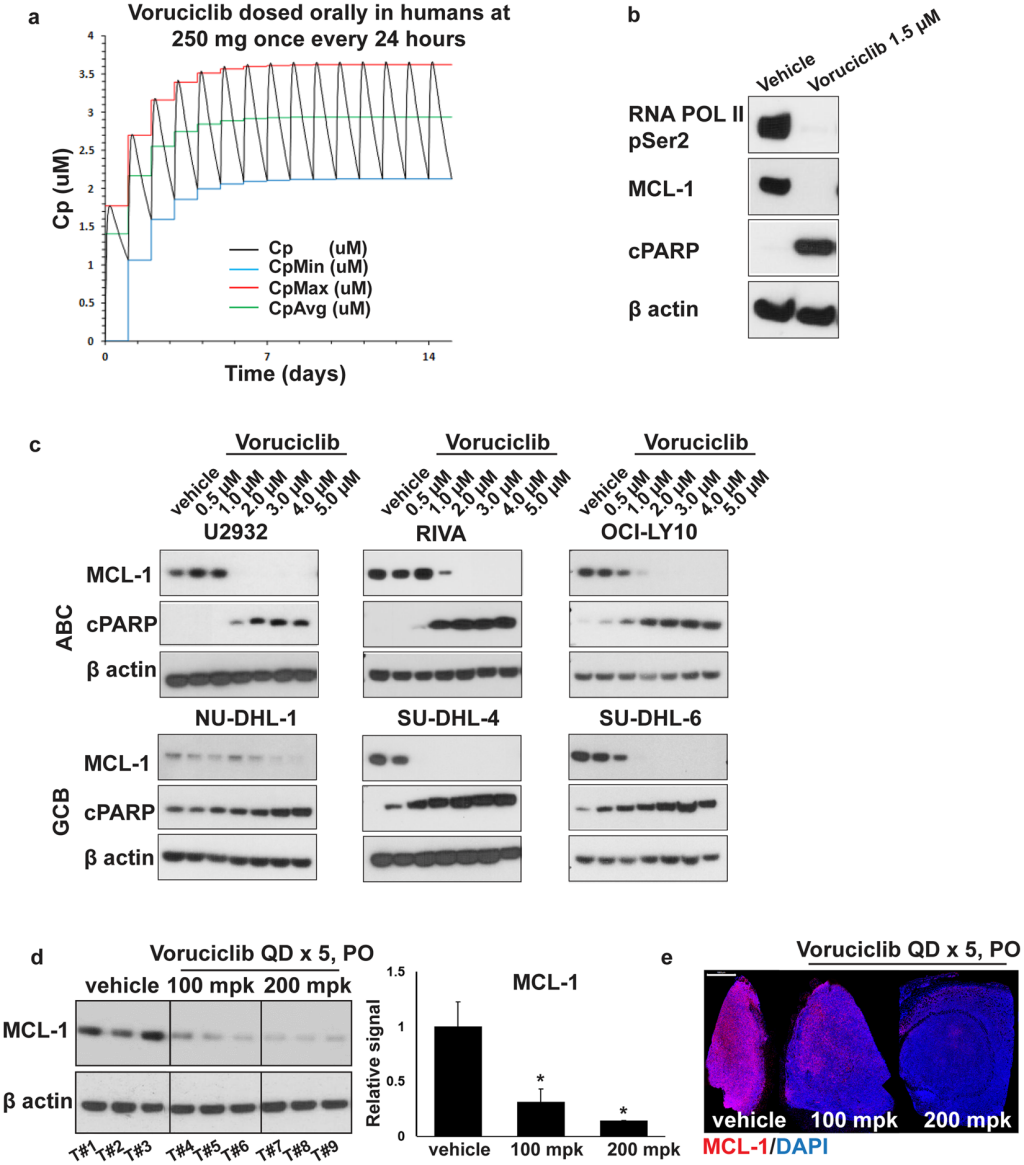
Voruciclib at clinically relevant concentrations leads to down regulation of MCL-1 protein **(a)** Predicted plasma concentrations in humans dosed daily orally with 250 mg voruciclib. Maximum, average and minimum plasma levels are shown by the red, green and blue lines respectively. The parameter set derived from the PK data after 13/15 days dosing was used for the 15-day simulation for the steady state levels attained after repeat dosing. **(b)** Western blot analyses for RNA POL II (pSer2), MCL-1, cPARP using protein lysates isolated from SU-DHL-4 cells. β actin was used as the loading control. **(c)** Western blot analyses for MCL-1 and cPARP from protein lysates isolated from U2932, RIVA, OCI-LY10 cells (ABC subtype) and NU-DHL-1, SU-DHL-4 and SU-DHL-6 cells (GCB subtype) treated with voruciclib (0.5 µM–5 µM) for 6 hours. β actin was used as the loading control. **(d**,**e**) Western blot and immunohistochemical (IHC) analyses for MCL-1, using tumor lysates and formalin-fixed paraffin-embedded sections respectively, obtained from OCI-LY10 xenograft bearing mice (n = 3 per condition) orally treated with vehicle, voruciclib 100 mpk or 200 mpk for 5 consecutive days. Tumors were resected 4 hours after the final dose and processed for both Western blot and IHC analyses. β actin was used as loading control for the Western blot where each lane represents an individual tumor (Tumor (T) #1-9). MCL-1 signals, normalized to β actin, were quantified and the average MCL-1 signal for each condition relative to vehicle is shown in the corresponding bar graph. Error bars represent standard error of the mean (SEM). Asterisks (*) denote *p* value < 0.05 calculated using the Student’s t-test for comparison of vehicle vs treatment. DAPI was used as counterstain for IHC. Representative images are shown. Scale bar: 1000 µm.

Since target profiling studies revealed CDK9 as a main target of voruciclib, we next assessed the impact of voruciclib on MCL-1 expression in cell-based and xenograft models of DLBCL. To understand whether clinically achievable and tolerated levels of voruciclib repress MCL-1, we used existing pharmacokinetic data from the two completed Phase 1 trials to model expected plasma drug levels achieved upon dosing with a conservative regimen of 250 mg/day voruciclib^26,27^. (Fig. 2a). Predicted mean plasma levels, once steady state is achieved after about 5 oral doses, fluctuate between 2 and 3.5 µM with an average near 3 µM for our model parameters. The concentrations of voruciclib used for cell-based investigations was set to encompass this range of plasma drug levels achieved following systemic exposure in humans. Consistent with its function as a CDK9 inhibitor, treatment with voruciclib led to inhibition of phosphorylation of RNA POL II, a well-characterized direct substrate of CDK9, and repression of MCL-1 expression in SU-DHL-4 cells (Figs 2b and S1). Moreover, Western blot analyses of lysates derived from 6 distinct DLBCL models representing both ABC and GCB subtypes treated with voruciclib showed targeted downregulation of MCL-1 in comparison to the respective vehicle controls (Figs 2c, S2 and S5). This was accompanied by apoptosis as represented by cleaved PARP induction starting at 1–2 μM in all models (Figs 2c, S2 and S5). Complete MCL-1 repression was typically observed at 3 μM exposures. Furthermore, since voruciclib has been shown to be orally bioavailable, we investigated whether MCL-1 repression could be detected in tumors of xenografted mice following administration of voruciclib by oral gavage. Consistent with effective *in vivo* CDK9 inhibition following oral delivery, MCL-1 protein expression was decreased in a dose-dependent manner, as observed upon comparing both tumor lysates and immunohistochemically stained sections from mice receiving either vehicle, 100 mpk or 200 mpk voruciclib daily for 5 days (Figs 2d,e and S1). Thus, voruciclib displays effective CDK9 activity with repression of MCL-1 both *in vitro* and *in vivo* following oral delivery in models of DLBCL.

### Combination of voruciclib and venetoclax leads to enhanced tumor growth inhibition compared to either drug alone, in specific models of DLBCL

**Figure 3:**
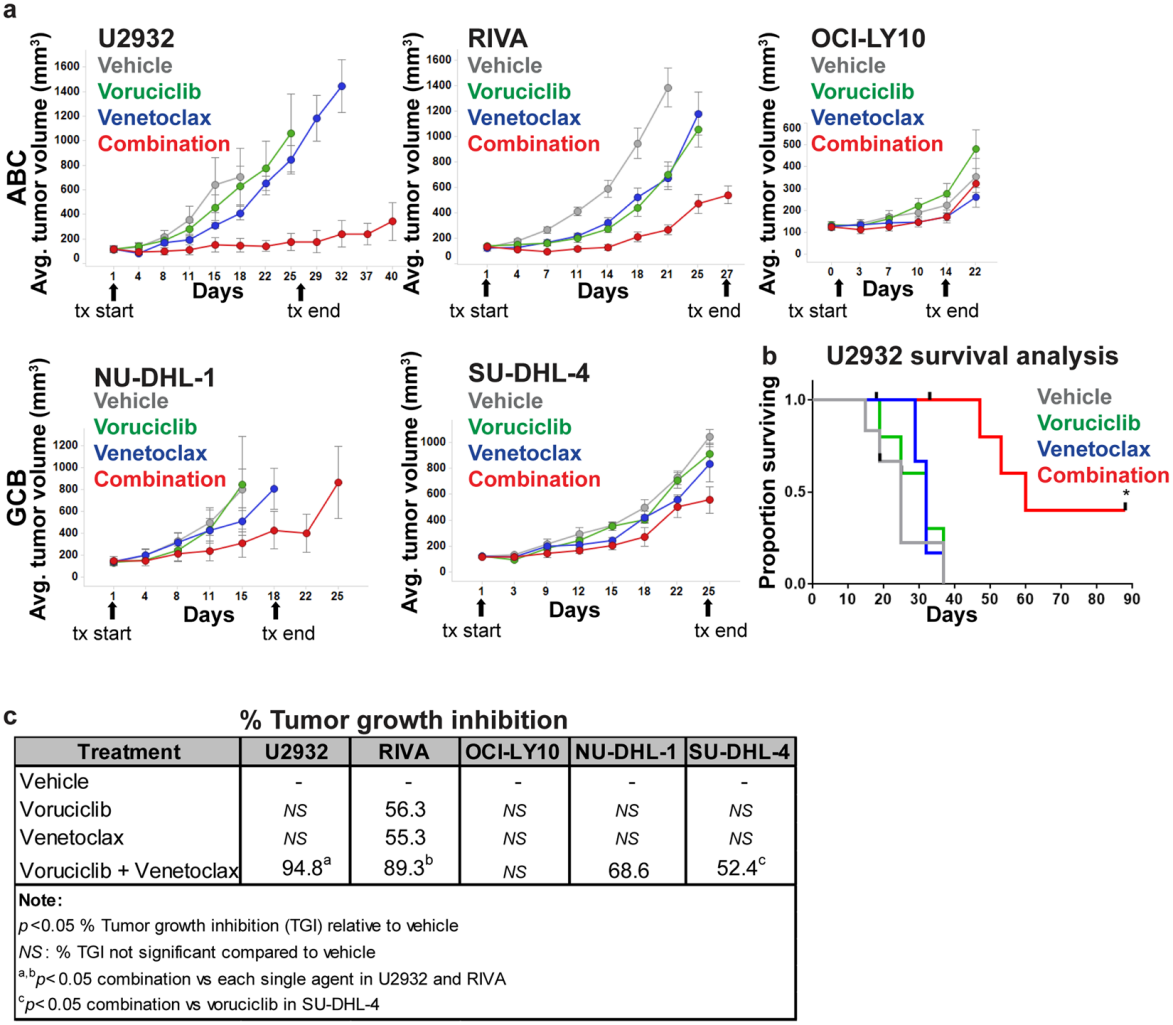
Voruciclib combined with venetoclax leads to model-dependent tumor growth inhibition and long term systemic benefits. **(a)** Tumor growth plots from U2932, RIVA, OCI-LY10, NU-DHL-1 and SU-DHL-4 xenografted mice (n = 5–6 per treatment condition for each model) respectively, orally treated with vehicles corresponding to both drugs (control arm), venetoclax, voruciclib or a combination of both drugs at the same dose and frequency as the corresponding single agents (see materials and methods for dosing regimens). Arrows indicate start and end day of treatment (tx). Drug efficacy with respect to vehicle was assessed in all arms via tumor volume measurements which are averaged across all subjects in the respective cohorts and plotted over time in days. Error bars represent SEM. **(b)** Kaplan Meier survival plots for each treatment cohort in the U2932 study are shown where xenografted mice were followed for up to 62 days after cessation of treatment. Asterisk (*) denotes *p* value < 0.05 calculated using the Log-rank test for comparison of combination versus single agent survival curves. **(c)** Percent tumor growth inhibition (TGI) relative to respective vehicle controls, were estimated using tumor volume measurements at Day 18 (U2932), Day 21 (RIVA), Day 22 (OCI-LY10), Day 18 (NU-DHL-1) and Day 25 (SU-DHL-4) (see methods). *p* values for TGI comparisons were calculated using the Mann-Whitney test.

Given that upregulation of MCL-1 has been documented as a mechanism of resistance to the BCL-2 specific inhibitor venetoclax, and that other CDK inhibitors with activity against CDK9 have been shown preclinically to enhance the anti-tumor effects of BCL-2 inhibition, we next assessed whether systemic administration of the combination of voruciclib and venetoclax causes significantly enhanced tumor growth inhibition versus exposure to either single agent alone. Both agents were administered by oral gavage. Efficacy, as measured by tumor growth inhibition (TGI) was assessed in 3 models of ABC (U2932, RIVA, and OCI-LY10) and two models of GCB (SU-DHL-4 and NU-DHL-1) subtype DLBCL to make an initial assessment of whether response to BCL-2/MCL-1 inhibition can be predicted based on genetic subtyping. To facilitate observation of drug combination effects, the dosing regimen of venetoclax was optimized for each model to reduce single agent antitumor efficacy. All regimens were well-tolerated with no overt signs of toxicity or body weight changes (Figure S6). The results of these studies were largely consistent with previous xenograft studies pairing venetoclax with other CDK9 inhibiting agents^29^. While anti-tumor responses to single agents were limited, the combination of voruciclib and venetoclax showed a range of effects across models (Fig. 3). The best response to the combination treatment was observed in the U2932 model where tumor remission was durable for more than 45 days after cessation of treatment (Fig. 3). A second model of ABC DLBCL, RIVA, exhibited the next best response to the drug combination with significantly higher tumor growth inhibition compared to subjects receiving either agent alone. Interestingly, a third model of ABC, OCI-LY10, did not respond to any treatment, while both GCB models exhibited modest but significant responses to the drug combination compared to vehicle controls (Fig. 3). These results suggest that the combination of voruciclib and venetoclax holds promise for treating a subset of patients with high risk ABC DLBCL and given the low response rates in this subtype to currently available treatment options, this merits consideration for clinical investigation.

### Multiplexed tumor microdosing studies indicate tumor-specific responses to combined exposure to voruciclib and venetoclax *in vivo*

**Figure 4:**
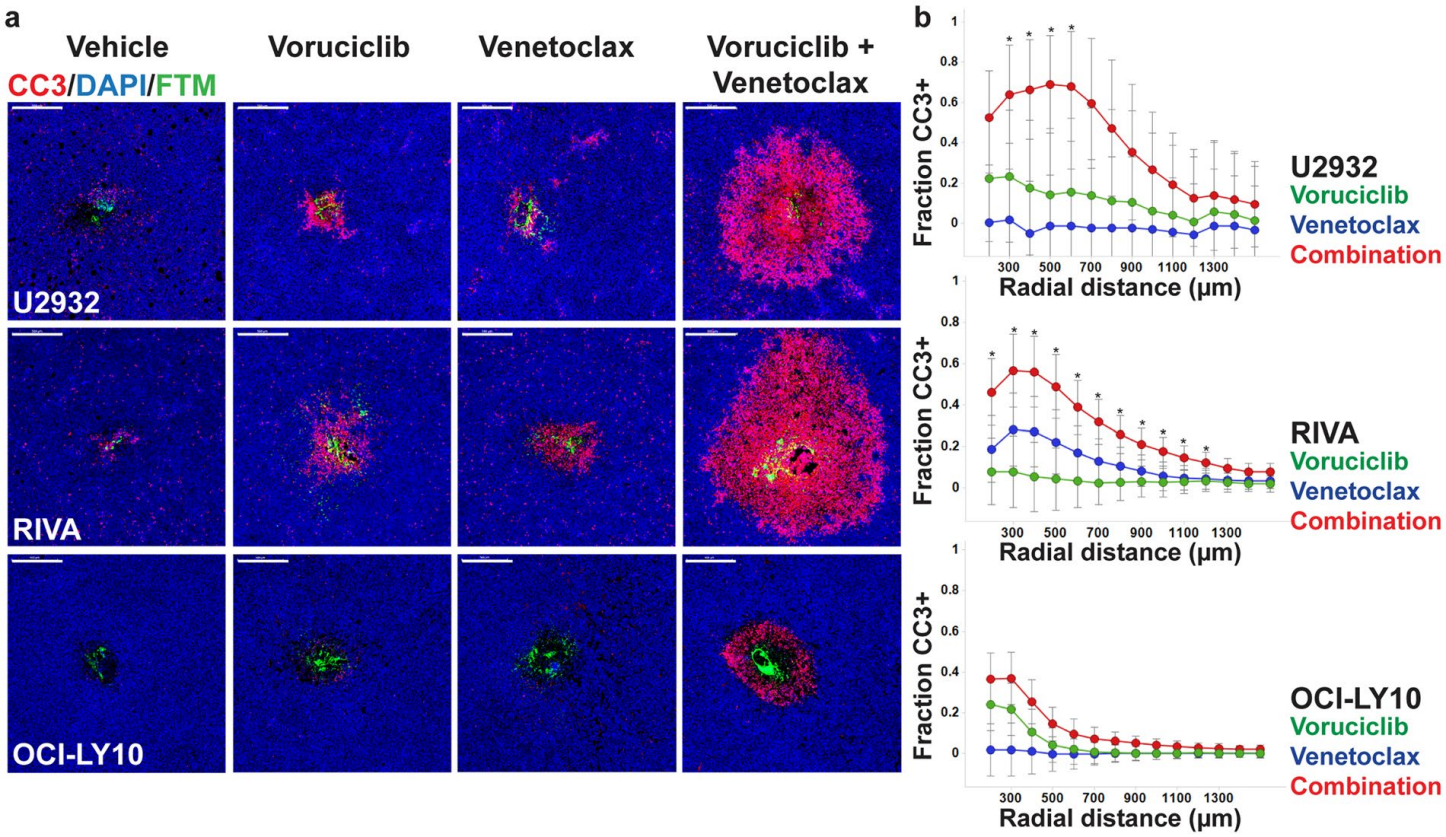
: Localized tumor apoptosis following drug microdosing with CIVO differentiates sensitive from resistant ABC DLBCL models, *in vivo*. **(a)** For combination analysis, U2932, RIVA, OCI-LY10 tumors (ABC subtype) were micro-injected with vehicle, voruciclib, venetoclax and voruciclib + venetoclax (U2932 n = 7; RIVA n = 12; OCI-LY10 n = 8 tumors) and resected 24 hours post injection. Fluorescent tracking marker (FTM) demarcates the sites of injection. Tissue sections were stained for cleaved caspase 3 (CC3) and DAPI. Representative images are shown. Scale bar: 500 µm **(b)** Radial effect plots show the difference between fraction CC3 + cells induced by voruciclib, venetoclax or the combination versus the vehicle control, as a function of radial distance from the site of injection demarcated by FTM. Error bars denote 95% confidence intervals. Asterisks (*) indicate statistical significance (*p* values < 0.05) at radial distances where CC3 responses induced by the combination are greater than both single agents.

The model-specific responses displayed by the ABC subtype tumors following exposure to voruciclib and venetoclax, while only observed in a subset of models, is in line with several recent studies indicating that prediction of tumor response based solely on genetic characterization is challenging, and that functional precision medicine methods are needed^30^. We have previously shown that CIVO arrayed microinjection technology (Presage Biosciences, Seattle WA) can be used to assess multiple drugs and drug combinations simultaneously in a single tumor to efficiently determine anti-tumor efficacy and potential synergy between drugs of interest^31,32^. We therefore assessed whether CIVO represents a viable functional precision medicine approach for identifying potential responders versus non-responders to voruciclib/venetoclax combination therapy. Towards this goal, the CIVO platform was used to simultaneously microinject either voruciclib, venetoclax, a combination of the two agents or vehicle control directly into spatially defined regions of xenografted tumors representing the three ABC models described above. Consistent with the potential to predict tumor responsiveness to the combination of voruciclib and venetoclax, both U2932 and RIVA models exhibited regions of robust enhancement of cleaved caspase 3 positive (CC3+) apoptotic cells that significantly surpassed responses induced by either single agent alone (Fig. 4). The corresponding *in vivo* combination indices in the U2932 and RIVA models were less than 1 (0.13 and 0.47 respectively) thereby denoting synergy^32^. Further suggesting that the combined effect of voruciclib and venetoclax is due to dual inhibition of MCL-1 and BCL-2, co-microinjection of selective MCL-1 inhibitor, A-1210477^33^ with venetoclax, induced similar enhancement of apoptosis (Figure S7). In contrast, localized apoptotic responses to voruciclib + venetoclax observed in OCI-LY10 tumors, were confined to the immediate region surrounding the site of microinjection and were not significantly different from single agent or control injections, consistent with observed systemic outcomes (Fig. 4). While an expanded analysis on a greater number of representative tumor models is required to ultimately assess the utility of CIVO as a *bona fide* predictive assay, these preliminary data are encouraging with regard to the capability of the CIVO platform to detect responsiveness to agents that interfere with the function of pro-survival BH3 targets.

### OCI-LY10 resistance correlates with drug-induced upregulation of BCL-xL

**Figure 5:**
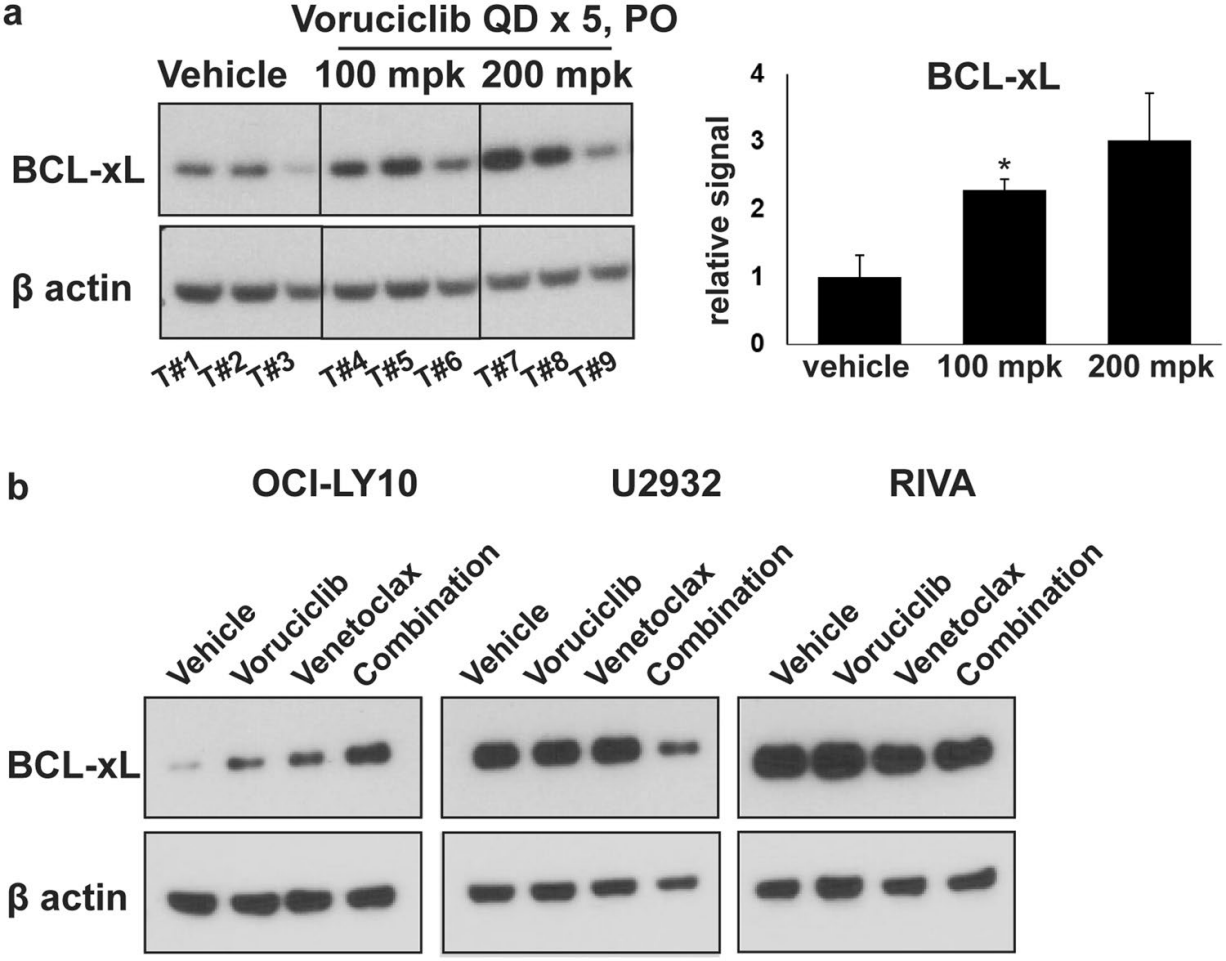
BCL-xL, a potential mediator of resistance, is upregulated in response to MCL-1/BCL-2 inhibition (**a**) Western blot analysis for BCL-xL protein using tumor lysates (previously described in Fig. 2d) derived from OCI-LY10 xenograft bearing mice (n = 3 per treatment) systemically treated with vehicle, voruciclib 100 mpk or 200 mpk by oral gavage for 5 consecutive days followed by tumor resections 4 hours after the final dose. β actin was used as the loading control. Each lane represents an individual tumor (Tumor (T) #1–9). BCL-xl signals, normalized to β actin, were quantified and the average BCL-xl signal for each condition relative to vehicle is shown in the corresponding bar graph. Error bars represent SEM. Asterisks (*) denote *p* value < 0.05 calculated using the Student’s t-test for comparison of vehicle vs treatment. **(b)** Western blot analyses for BCL-xL protein using lysates derived from ABC cell lines (OCI-LY10, U2932 and RIVA) treated with vehicle, voruciclib 5 µM (all lines), venetoclax (OCI-LY10 and U2932: 50 nM, RIVA 10 nM) or combination thereof, for 24 hours. β actin was used as the loading control.

The well-established functional redundancy between pro-survival BH3 members raises the possibility that BCL-xL mediates resistance to agents that specifically inhibit BCL-2 and MCL-1. We therefore investigated whether BCL-xL may play a role in the resistance to the combination of voruciclib and venetoclax as observed in the OCI-LY10 tumors. Consistent with this possibility, OCI-LY10 tumor lysates from mice subjected to oral gavage with voruciclib (as described previously in Fig. 2d), exhibited elevated levels of BCL-xL compared to vehicle controls as determined by Western blot analysis (Figs 5a and S8). Furthermore, lysates from OCI-LY10 cells showed elevated levels of BCL-xL in response to *in vitro* exposure to either voruciclib or venetoclax, with further enhancement when exposed to the combination of both drugs (Fig. 5b and S9). Of the three ABC models evaluated in this study, the observed drug-induced elevation of BCL-xL was unique to OCI-LY10 and not observed in U2932 or RIVA models. Interestingly, a modest downregulation of BCL-xL was observed in response to the combination in U2932 cells, the model most sensitive to combination treatment *in vivo*. While still correlative at this point, our data suggest that inhibition of BCL-xL along with inhibition of BCL-2 and MCL-1 may be required for inducing response in a subset of patients with high-risk lymphomas.

## Discussion

The frequent observance of resistance and relapse to first line R-CHOP therapy by ABC DLBCL underscores the need for novel approaches to treat this disease. Here we present a potential all-oral combination regimen consisting of a novel CDK9 inhibitor, voruciclib and the BCL-2 specific inhibitor, venetoclax, with promise for treating this high-risk subtype of DLBCL. While the combinations of venetoclax with other CDK9 inhibitors such as flavopiridol or dinaciclib have previously been proposed, neither of these two agents are orally bioavailable^34,35^. In addition to the convenience of an oral regimen, we demonstrate that voruciclib exhibits a more CDK-selective target profile compared to flavopiridol, which may reduce unintended off-target mediated adverse effects.

Voruciclib addresses a well-characterized key-mediator of resistance to venetoclax by repressing MCL-1. Consistent with the necessity for dual inhibition of functionally redundant pro-survival BH3 family members BCL-2 and MCL-1, combined administration of voruciclib and venetoclax resulted in significantly enhanced tumor growth inhibition compared to either single agent alone, in two distinct xenograft models of ABC DLBCL. Given that voruciclib has completed two Phase 1 clinical trials, it may represent the most clinically advanced orally available therapy to inhibit MCL-1 for use in combination with venetoclax to treat high-risk hematological malignancies.

The greater CDK selectivity of voruciclib compared to flavopiridol may have clinical implication and deserves further study. It is well-documented that the clinical utility of flavopiridol is hampered by dose-limiting toxicities including severe diarrhea (82% all grade, 18% grade 3 and above)^35^. While both compounds show comparable CDK9 activity, flavopiridol shows significantly higher affinity towards other non-CDK targets, notably ICK and MAK. ICK, originally cloned from the intestinal crypt, is ubiquitously expressed in adult human tissue but prominently throughout the intestinal tract and plays a role in cilia formation and signaling^36,37^. The importance of this kinase is underscored by the fact that a single loss-of-function mutation in this gene leads to a lethal, multi-organ disorder^38^. Similarly, MAK, also expressed in the intestine, has been shown to be important for regulation of ciliary length^36,39^. Therefore, it is reasonable to speculate that inhibition of such developmentally important, high-affinity non-CDK targets may contribute to the challenging toxicity profile of flavopiridol in the clinic. On the other hand, the lack of potent inhibition of these same targets by voruciclib may result in improved tolerability. Consistent with this, only one patient out of 24 (4%) treated at 350 mg/day voruciclib experienced dose-limiting diarrhea^26^. Since clinical investigation of voruciclib has thus far been limited to the solid tumor clinic, an expanded investigation into hematopoietic malignancies is warranted to assess whether an improved therapeutic window versus flavopiridol is observed in this context.

The therapeutic window for the voruciclib/venetoclax combination may also be improved by a functional precision oncology approach to identify patients who would benefit most from this treatment^40^. For inhibitors of more classic kinase targets such as B-Raf or the EGF receptor, the presence of activating mutations as identified by gene sequencing is used to define responders to the respective inhibitors. However, for agents targeting BH3 protein regulation of apoptosis, clear genomic predictors of responses are unlikely to be identified. The regulation of apoptosis is mediated by a complex interplay between multiple types of BH3-containing proteins at the mitochondrial membrane. These include effector molecules (BAX and BAK), pro-survival/anti-apoptotic proteins (BCL-2, MCL-1, BCL-xL, BCL-W, and BFL-1), pro-apoptotic sensitizer proteins (BAD, NOXA, PUMA, HRK, BMF, and BIK), and activator proteins (BIM and BID)^9,41–43^. The threshold for a tumor cell to undergo apoptosis is therefore not solely regulated by the expression level or activity state of any single BH3 protein, but dependent on the ratio of the entire family. Given that the two strongest responders in our *in vivo* study belong to the same genetic subtype as the model showing no response, this is clearly applicable for sensitivity to agents that directly or indirectly target BH3 family members. Several functional precision approaches are in development that will be useful for stratifying patients for BH3 targeting therapies. The most advanced to date is the method of dynamic BH3 profiling, in which patient cells are primed for apoptosis by introduction of different BH3 sensitizer proteins, thus revealing specific sensitivities to BCL-2 and MCL-1 targeting agents^41^. Here we demonstrate that arrayed intra-tumoral microinjection of drug micro-doses leads to localized responses to the combination of voruciclib and venetoclax that correlate with systemic responses to the same drug combination. Implementation of CIVO as a *bona fide* method of functional precision medicine will ultimately require prospective evaluation in the human cancer clinic. However, given that CIVO has already been investigated in lymphoma patients with cancerous, palpable lymph nodes, with no serious adverse effects^31^, application of an arrayed microinjection approach toward the identification and enrichment of responders to voruciclib/venetoclax combination therapy seems feasible for future clinical trials.

In conclusion, in this study we demonstrate that inhibition of two master regulators of cell survival, MCL-1 and BCL-2 with clinical stage drugs, voruciclib and venetoclax respectively, can achieve synergistic anti-tumor efficacy in a subset of ABC DLBCL models. High-risk ABC subtype of DLBCL remains an unmet clinical need despite improvements in DLBCL treatment outcomes in the rituximab era^3,44^. Potential new therapies for high-risk DLBCL, such as the one described here would potentially benefit from the development of functional precision oncology methods like CIVO that complement existing genomic methods to guide personalized cancer treatment. Together, future clinical investigations of this combination along with validated precision oncology approaches may pave the way for a novel treatment strategy for high-risk lymphoma patients.

## Materials and Methods

### DiscoveRx and Thermofisher screens

Percent inhibition of kinase activity for 468 kinase assays of the DiscoveRx ScanMax kinase panel and 414 kinase assays of the ThermoFisher SelectScreen kinase panel at 10 µM and 50 nM were carried out with voruciclib and flavopiridol.

### Reaction Biology IC50 profiling

Rank order of sensitivity of 48 kinases to voruciclib hydrochloride was determined at Reaction Biology Corp. Kinase activity was measured using a filter binding assay with radioactive γ-33P-ATP as phosphate donor. The ATP concentration was near the K_m_ values for the respective kinases. For each kinase, an IC50 value was calculated from a 10-point concentration curve of the test article and converted to K_i_ values. The 48 kinases studied here had been identified in previous screening experiments as the most promising target candidates.

### Cell culture

RI-1, also known as RIVA, U2932, NU-DHL-1 cell lines were purchased from DSMZ. SU-DHL-4, SU-DHL-6 were purchased from ATCC. All lines were cultured in RPMI 1640 with L-Glutamine (ThermoFisher),10% fetal bovine serum (Thermofisher), at 37 degrees Celsius, 5% CO_2_. OCI-LY10 cells, obtained from University Health Network, Ontario, Canada, were cultured in Iscove’s Modified Dulbecco’s medium (Thermofisher) with 20% human serum (Valley Biomedical). IMPACT III testing (IDEXX Bioresearch) was carried out on cell lines to confirm mycoplasma- and pathogen-free status. Cell lines were expanded and cryopreserved following 2–3 passages after obtaining from vendor. After thawing, cells were maintained (for experiments, xenografting) for a maximum of 8 weeks by sub-culturing approximately 3 times a week.

### Western Blot Analysis

20 micrograms of protein lysate from each condition were subjected to SDS-PAGE and transferred to 0.45 micron PVDF membranes (ThermoFisher) using manufacturer’s protocol, blocked for one hour in 5% milk at room temperature, probed with primary antibodies for cPARP (rabbit CST 5625), MCL-1 (rabbit CST 39224) or BCL-xL (rabbit CST 2764), followed by corresponding HRP-conjugated secondary antibodies (Jackson ImmunoResearch). β actin (mouse CST 12262) was used as loading control. ECL chemiluminescent substrate (Pierce) and autoradiographic film (ThermoFisher) were used for detection of signals. MCL-1 and BCL-xL signals were quantified using Image Studio Lite (LICOR Biosciences) using β actin for normalization. Student’s t-test was used to compare mean signal intensities across different conditions. The level of significance was set at 0.05.

### ***in vivo*studies**

All experiments in mice were approved by IACUC Board of Presage Biosciences, Seattle, WA (Protocol number PR-001) and were performed in accordance with relevant guidelines and regulations. All relevant procedures were performed under anesthesia and all efforts were made to minimize pain and suffering. None of the mice contributing to this study became ill or died prior to experimental endpoints and all mice receiving drug treatment as described below, underwent routine health monitoring and were humanely euthanized. Female NOD.CB17-Prkdcscid/NCrHsd mice (Envigo) with an average weight of 22 gms were used for experiments between 5–15 weeks of age. Subcutaneous flank xenografts were generated with U2932, RIVA, OCI-LY10, NU-DHL-1 and SU-DHL-4 cell lines. Tumors were generated by right flank injection of 5 × 10^6^ cells in 200 µl cell suspension of Matrigel (Corning) and serum free media in 1:1 ratio per mouse. Mice were enrolled into arrayed microinjection studies when the xenografted tumor volume reached approximately 1000 mm^3^ as previously described^31^. Briefly, the CIVO device was configured with 6 injection needles set for a 6 mm injection length and a total volume delivery of 3 μl. A fluorescent tracking marker (FTM) was added to each drug or drug combination. All micro-doses were equivalent to or lower than what would be allowed under FDA guidelines for Exploratory IND (Investigational New Drug) studies and by solubility of drug into vehicle. Total amounts of agents injected were 15.2 µg of voruciclib (Presage), 26 ng of venetoclax (Chemietek) and 6.4 µg of A-1210477 (Selleck).

For systemic drug efficacy studies, mice were enrolled when tumors reached an average volume of 150–200 mm^3^. Assignment to treatment groups was carried out via stratified randomization^45^. Tumor dimensions were measured twice a week using digital calipers along with recording of body weight. Tumor volume was calculated using formula *V* = π/6 (length x width x height) in mm^3^ ^46^. Animals were removed from the study when either any one of the three measured dimensions of the tumor exceeded 2 cm, volume exceeded 2000 mm^3^, ulceration was observed or if body weight loss greater than 20% was recorded. Drug formulations: voruciclib was formulated in 0.1% methylcellulose (Sigma) and venetoclax in 60% phosal 50, 30% PEG 400, 10% ethanol^47^, both administered orally. Control cohorts were administered vehicles of both drugs, and each single agent cohort was administered the vehicle of the other drug. Depending on the assigned treatment, venetoclax (or its vehicle) was followed by voruciclib (or its vehicle) with 30 mins in between administrations to allow for gastric clearance as per veterinary recommendation. Model specific dosing regimens: U2932: venetoclax at 10 mpk twice a week, voruciclib 200 mpk six days per week for a total duration of 4 weeks; RIVA: venetoclax at 1 mpk twice a week, voruciclib 200 mpk six days per week for a total duration of four weeks; OCI-LY10: venetoclax at 25 mpk twice a week, voruciclib 200 mpk six days per week for a total duration of two weeks; NU-DHL-1: venetoclax at 50 mpk once a week, voruciclib at 200 mpk five days per week for a total duration of three weeks; SU-DHL-4: venetoclax at 25 mpk and voruciclib 200 mpk both six days per week for a total duration of four weeks. The venetoclax + voruciclib combination treatment cohort for each model received both drugs at the same doses and frequencies as the corresponding single agents.

Tumor Growth Inhibition (TGI) % with respect to vehicle was calculated using following formula at the last time point before any mouse had to be removed from study for reasons stated above.

$$\frac{({V}_{final}({\rm{vehicle}})-{V}_{initial}({\rm{vehicle}}))-({V}_{final}({\rm{treatment}})-{V}_{initial}({\rm{treatment}}))\,}{({V}_{final}({\rm{vehicle}})-{V}_{initial}({\rm{vehicle}}))}\times 100$$

Volume measurements were averaged across tumors in respective treatment arms. Mann-Whitney test was used to determine statistical significance of TGI differences. Log-rank test was used to determine statistical significance of differences between Kaplan-Meier survival curves (GraphPad Prism). The level of significance was set at 0.05.

### Tissue processing, gross tissue imaging and immunohistochemistry

Following resections of CIVO microinjected tumors, tissue was fixed, processed, stained and scanned as previously described^31^. Rabbit anti-CC3 antibody (Cell Signaling #9661, 1:150 dilution) and rabbit anti-MCL-1 antibody (Cell Signaling #5453, 1:500 dilution) were used for IHC analysis. For immunofluorescent detection, secondary antibody conjugated to AlexaFluor647 (Jackson Immuno research, 1:600 dilution) was applied as per manufacturer’s instructions and tissues were counterstained with DAPI.

### Statistical approach for *in vivo* drug synergy analysis

Quantitative analyses of drug responses were performed using custom software (CIVOanalyzer, Presage Biosciences, Seattle) as previously described^31,32^. This software allows the automatic identification of injection sites with the help of the fluorescent tracking markers. Then, within circular regions of interest (ROIs) centered on the injection sites, cells are automatically identified and assigned to different classes distinguished by their staining patterns. The fraction of cells belonging to each class (e.g. CC3 positive) are mapped as a function of radial distance (a surrogate for local concentration) to obtain the radial effect curve f(r).

For two drugs administered in combination using the CIVO device, synergy evaluation was carried out as previously described^32^. Briefly, the radial effect curves for each agent alone, their combination and a vehicle control are used in conjunction with a linear mixed effects model to estimate whether the combination effect is greater than a linear combination of individual agent effects and hence synergistic. In addition, the model is used to determine the *in vivo* combination index based on Chou-Talalay analysis^48^. The level of significance was set at 0.05.

### Data availability statement

The datasets generated during and/or analyzed during the current study are available from the corresponding author on reasonable request.

## Electronic supplementary material

Supplementary figures
