# Supplementary material for: Voruciclib, a clinical stage oral CDK9 inhibitor, represses MCL-1 and sensitizes high-risk Diffuse Large B-cell Lymphoma to BCL2 inhibition
Source: Sci Rep. 2017 Dec 21;7:18007. doi: 10.1038/s41598-017-18368-w (PMC5740070; doi:10.1038/s41598-017-18368-w)

Title Page

Supplementary Information File

Voruciclib, a clinical stage oral CDK9 inhibitor, represses MCL-1 and sensitizes high-risk Diffuse Large B-cell Lymphoma to BCL2 inhibition

Joyoti Dey[1], Thomas L. Deckwerth[1], William S. Kerwin[1], Joseph R. Casalini[1], Angela J. Merrell[1], Marc O. Grenley[1], Connor Burns[1], Sally H. Ditzler[1], Chantel P. Dixon[1], Emily Beirne[1], Kate C. Gillespie[1], Edward F. Kleinman[2], Richard A. Klinghoffer[1]

[1]Presage Biosciences, Inc. Seattle, WA, USA
[2]Edward F. Kleinman LLC, Pawcatuck, CT

Corresponding author: Richard Klinghoffer (rich.klinghoffer@presagebio.com)

Supplementary Figure Legends

Figure S1

Full-length blots for western blot experiment in the SU-DHL-4 cell line for markers RNA POL II (pSer2), MCL-1, cPARP and β actin presented in Figure 2b and for experiment using OCL-LY10 xenograft tumor lysates presented in Figure 2d for markers MCL-1 and β actin.

Figure S2

Full-length blots for western blot experiment in the U2932 cell line for markers MCL-1, cPARP and β actin presented in Figure 2c

Figure S3

Full-length blots for western blot experiment in the RIVA cell line for markers MCL-1, cPARP and β actin presented in Figure 2c.

Figure S4

Full-length blots for western blot experiment in the NU-DHL-1, SU-DHL-6 and OCI-LY10 cell lines for markers MCL-1, cPARP and β actin presented in Figure 2c.

Figure S5

Full-length blots for western blot experiment in the SU-DHL-4 cell line for markers MCL-1, cPARP and β actin presented in Figure 2c.

Figure S6

Systemic administration of voruciclib, venetoclax or a combination thereof, did not induce any significant body weight changes

Plots represent body weight of mice recorded over the first two cycles of treatment in the U2932, RIVA, OCI-LY10, NU-DHL-1 and SU-DHL-4 xenograft models. Data are averaged across all tumors in the respective cohorts. Error bars represent SEM.

Figure S7: Voruciclib or A-1210477 combined with venetoclax have similar effects on tumor cell apoptosis *in vivo*: U2932 and RIVA tumors (ABC subtype) were micro-

injected with voruciclib, A-1210477, venetoclax, voruciclib + venetoclax and A-1210477 + venetoclax and resected 24 hours post injection. Fluorescent tracking marker (FTM) demarcates the sites of injection. Tissue sections were stained for cleaved caspase 3 (CC3) and DAPI. Representative images are shown. Scale bar: 500 µm

Figure S8

Full-length blots for western blot experiment using OCL-LY10 xenograft tumor lysates, for markers BCL-xL and β actin, presented in Figure 5a.

Figure S9

Full-length blots for western blot experiment in U2932, RIVA and OCI-LY10 cell lines, for markers BCL-xL and β actin presented in Figure 5b.

Figure S1

Western blot raw data pertaining to Figure 2b

After Black and White adjustment | *Corresponding original film scans*

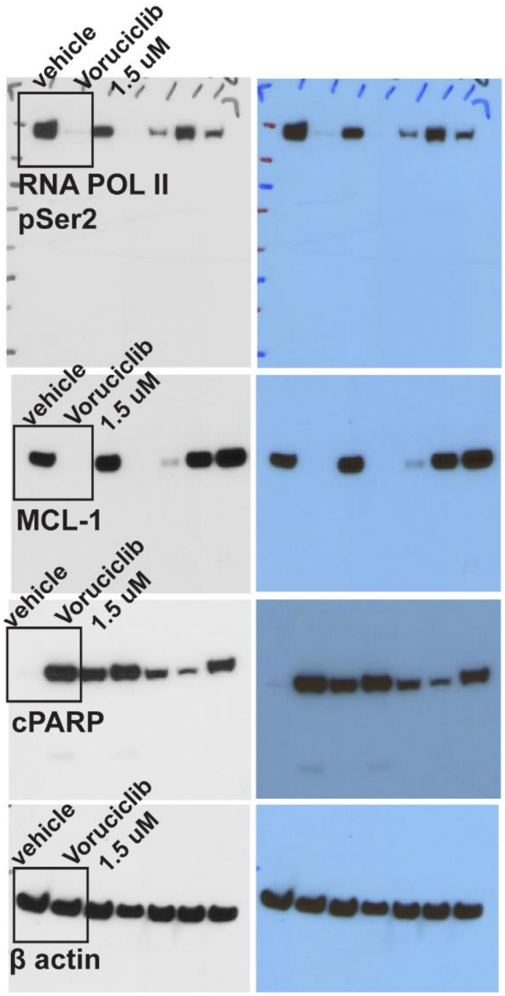

Western blot raw data pertaining to Figure 2d

After Black and White adjustment | *Corresponding original film scans*

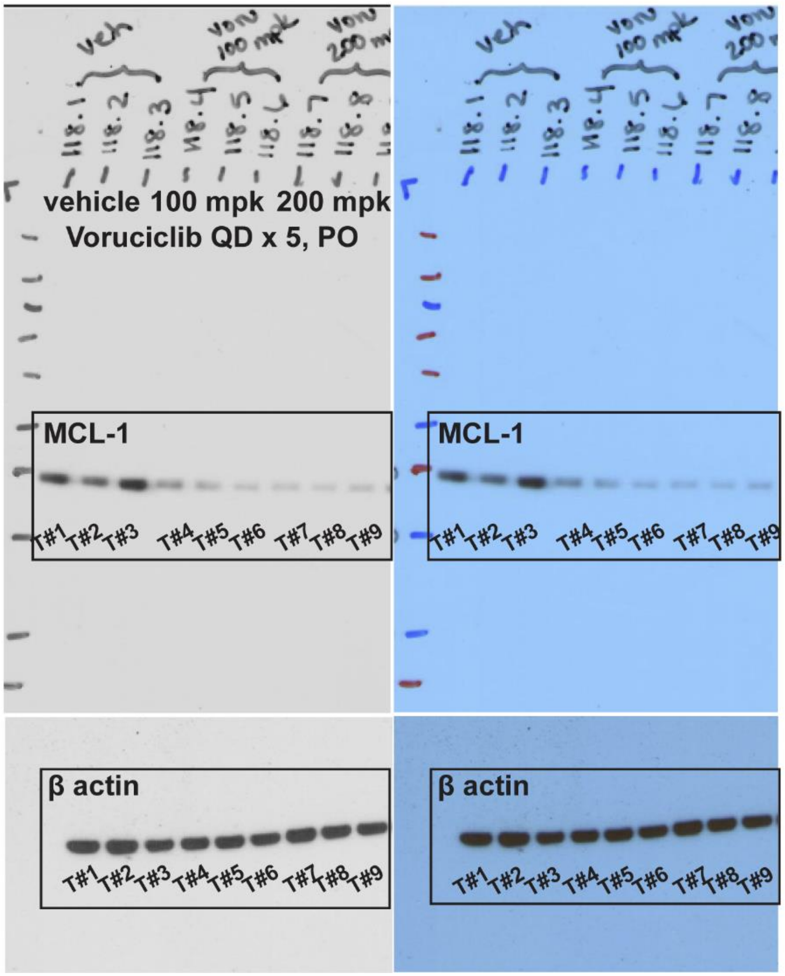

Figure S2

U2932 Western blot raw data pertaining to Figure 2c

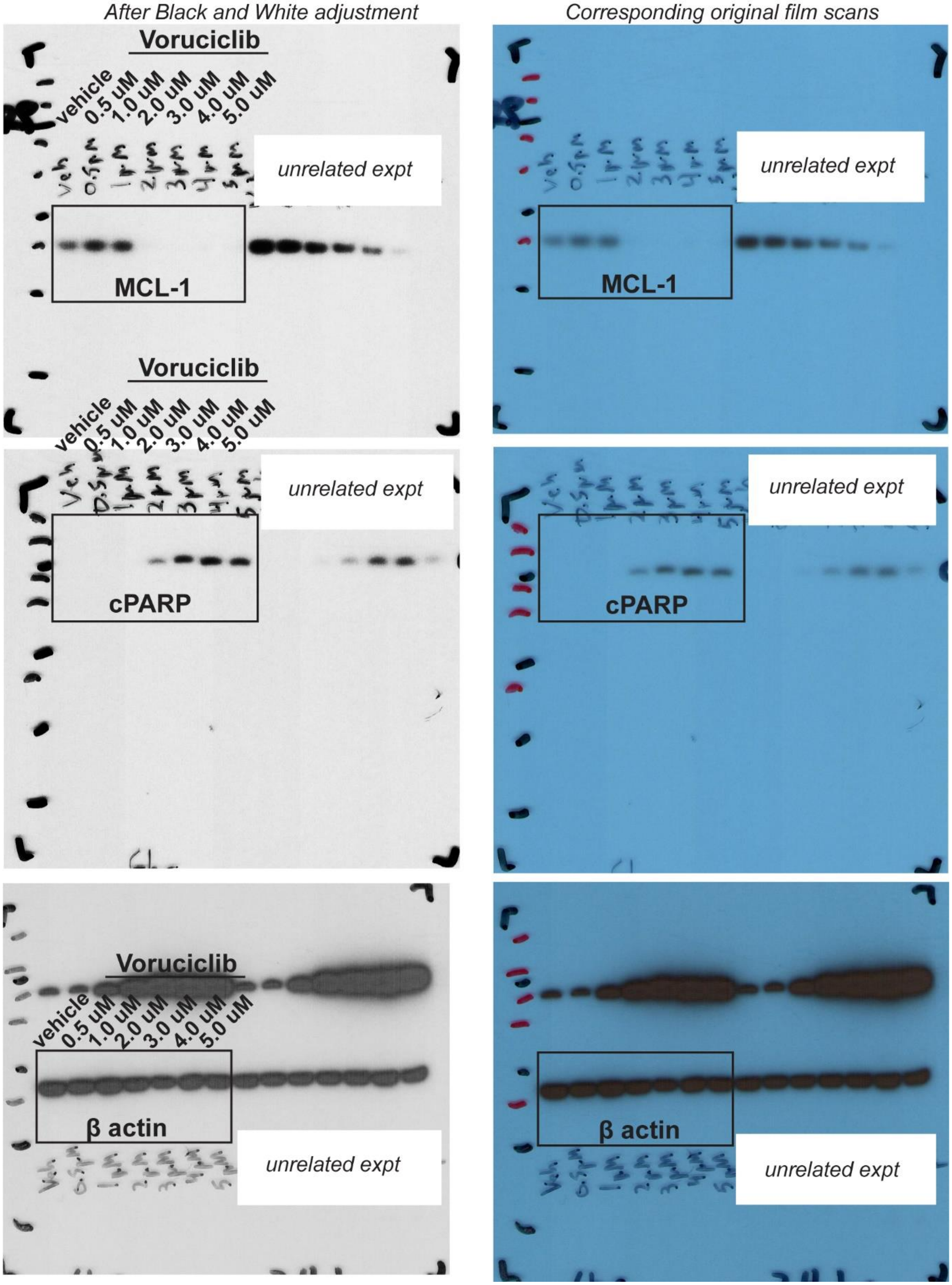

Figure S3

RIVA Western blot raw data pertaining to Figure 2c

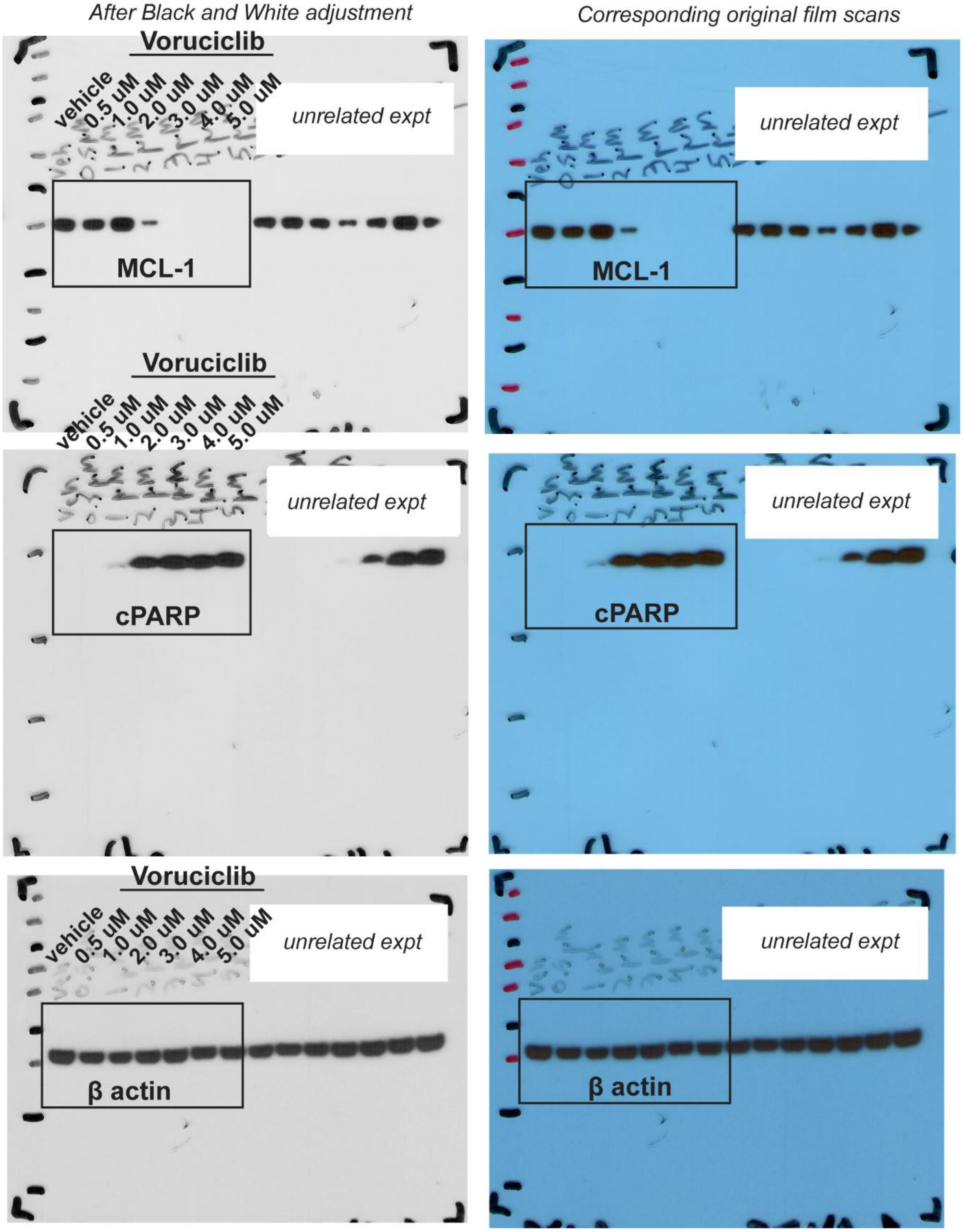

Figure S4

NU-DHL-1, SU-DHL-6, OCI-LY10 Western Blot Raw data pertaining to Figure 2c

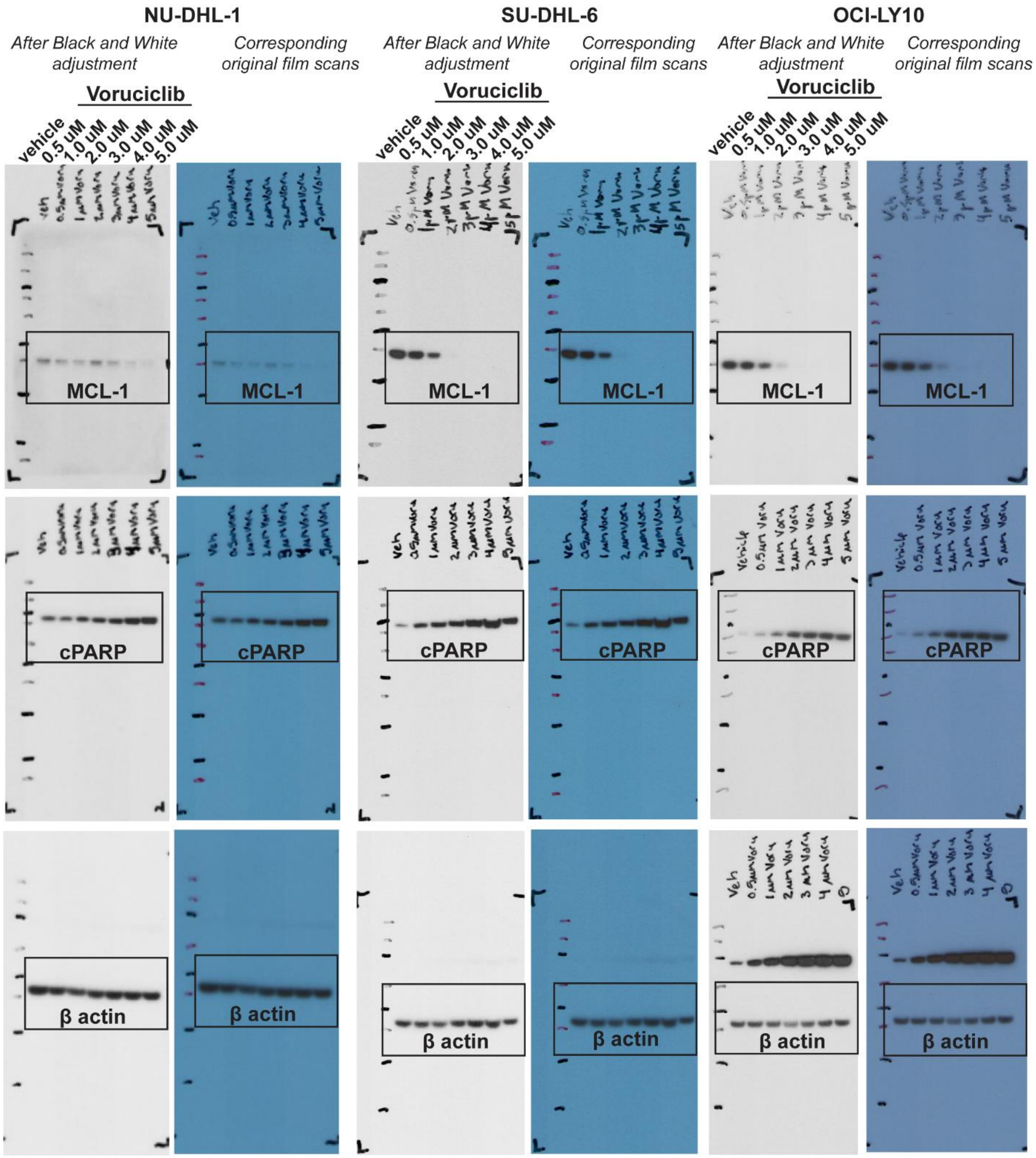

Figure S5

SU-DHL-4 Western Blot Raw data pertaining to Figure 2c

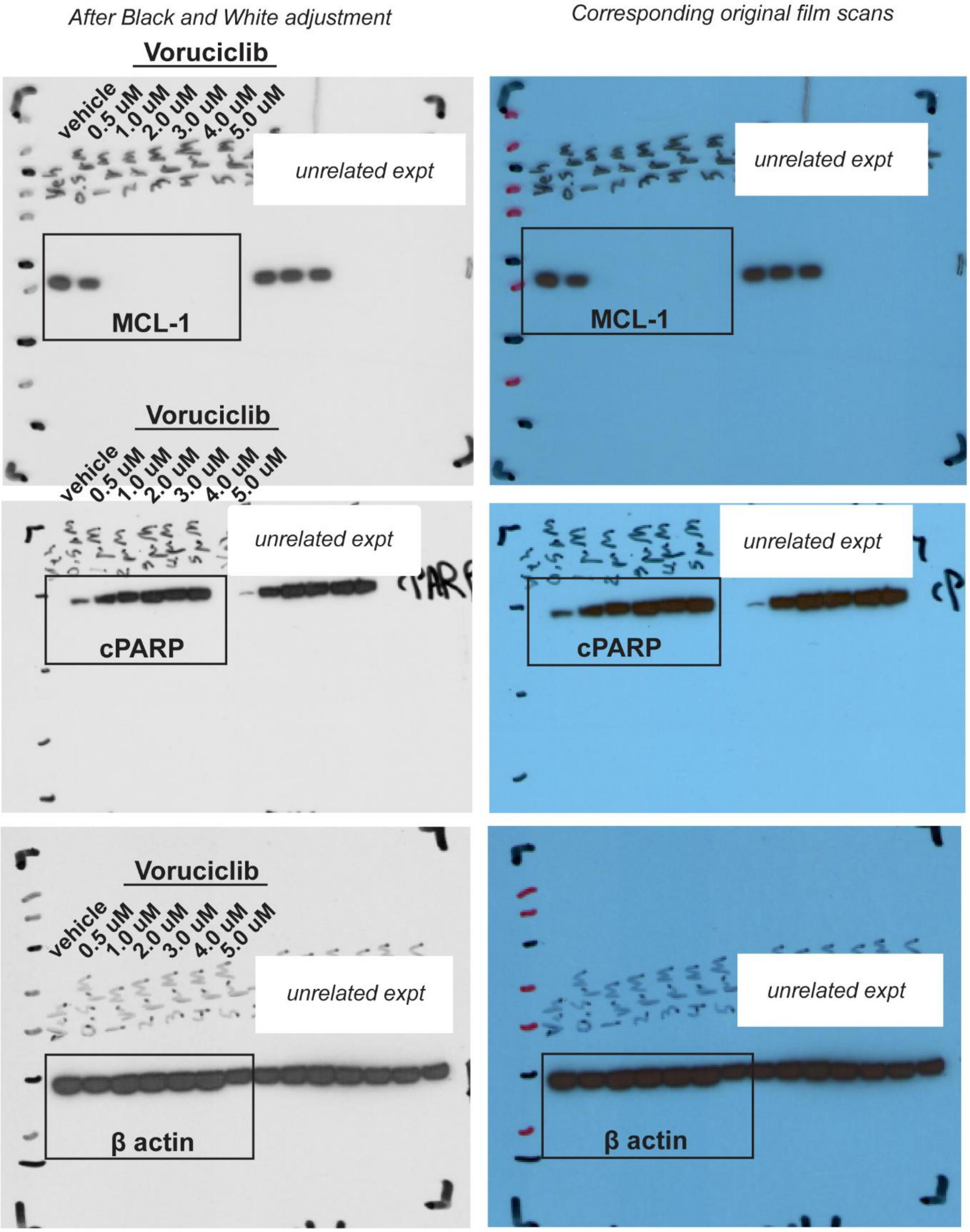

Figure S6

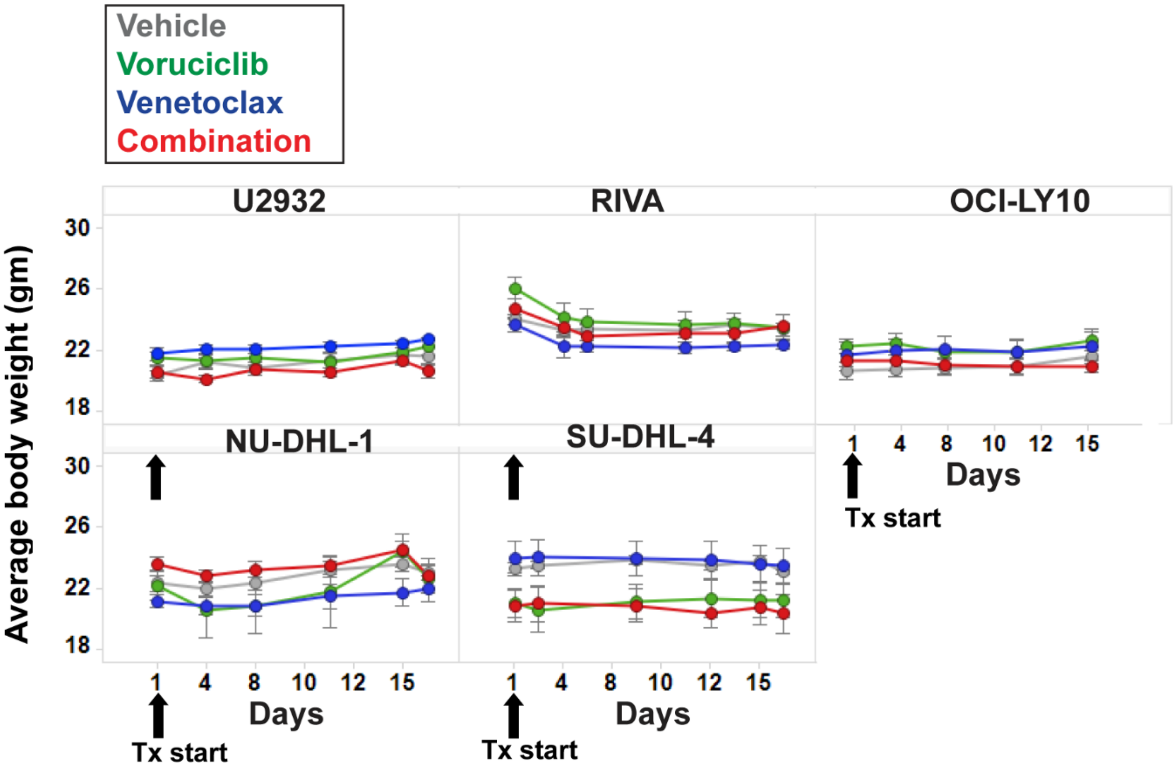
Vehicle
Voruciclib
Venetoclax
Combination
U2932
RIVA
OCI-LY10
NU-DHL-1
SU-DHL-4
Average body weight (gm)
30
26
22
18
1
4
8
10
12
15
Days
Tx start

Figure S7

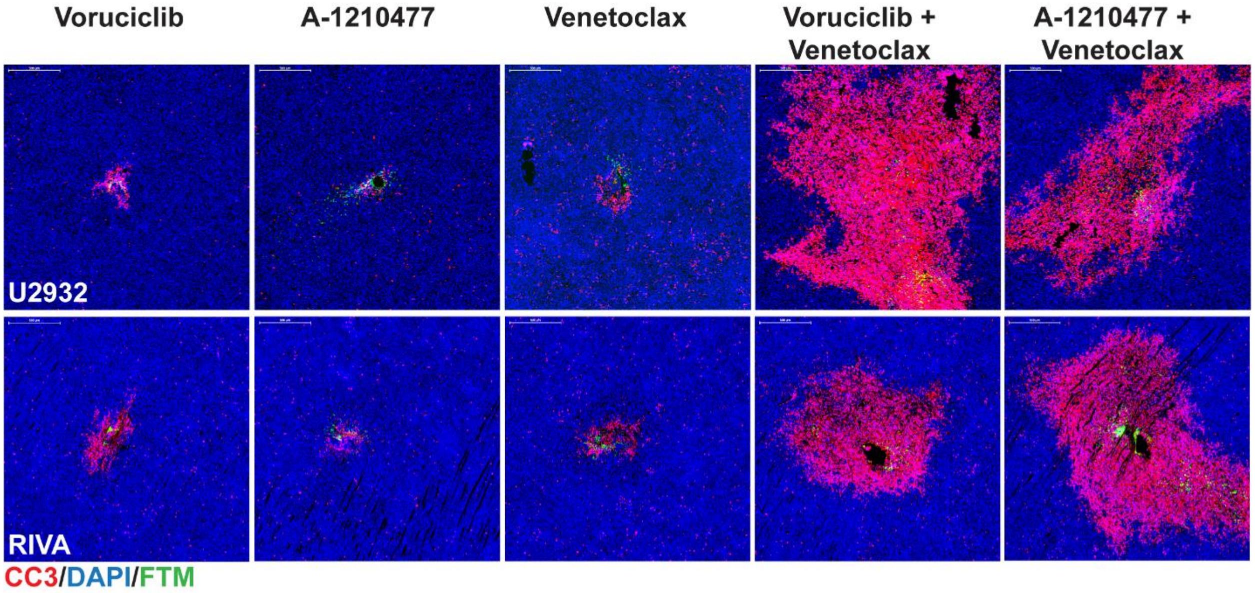
Voruciclib
A-1210477
Venetoclax
Voruciclib + Venetoclax
A-1210477 + Venetoclax
U2932
RIVA
CC3/DAPI/FTM

Figure S8

Western Blot Raw data pertaining to Figure 5a

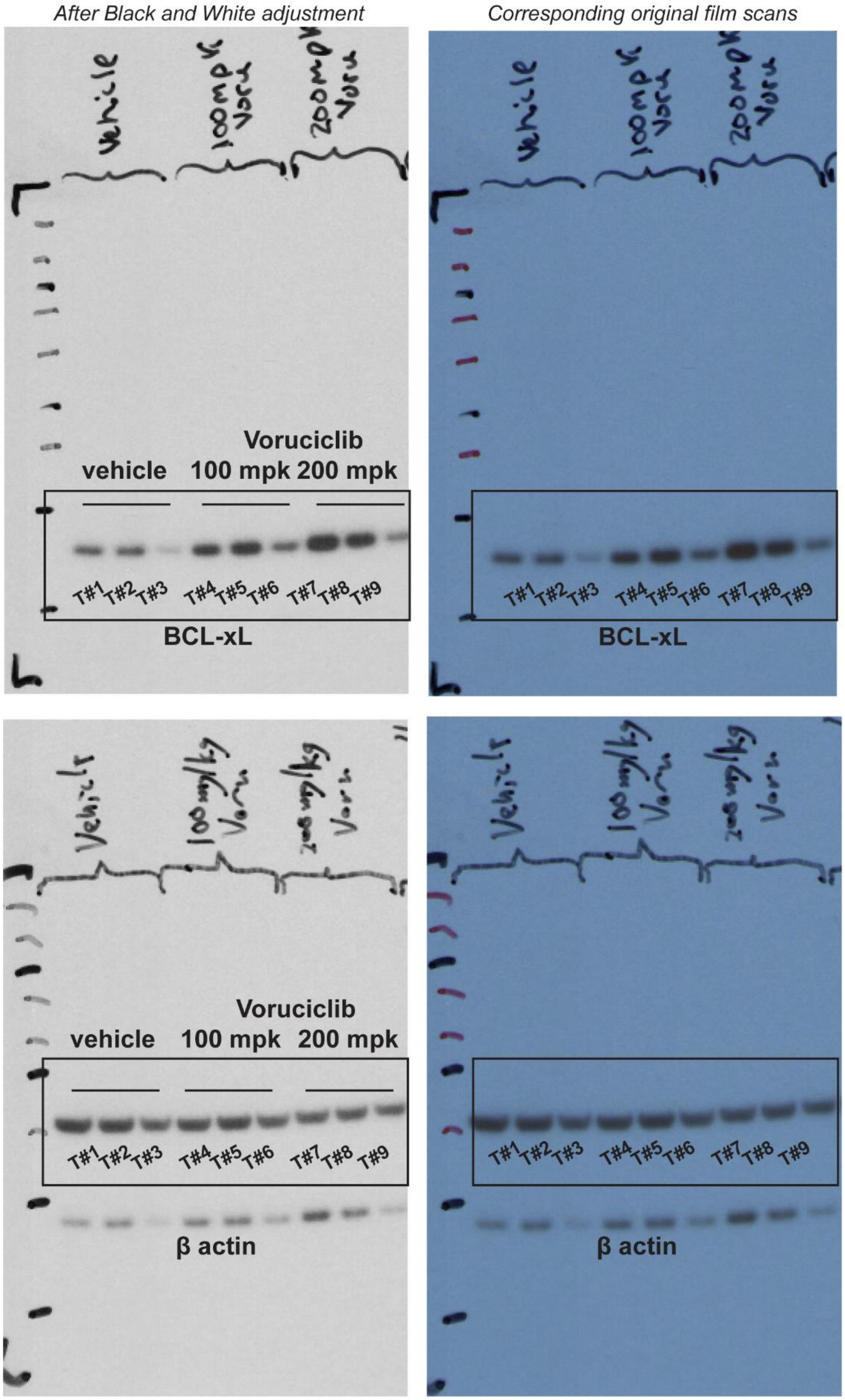

Figure S9

Western Blot Raw data pertaining to Figure 5b

After Black and White adjustment *Corresponding original film scans*

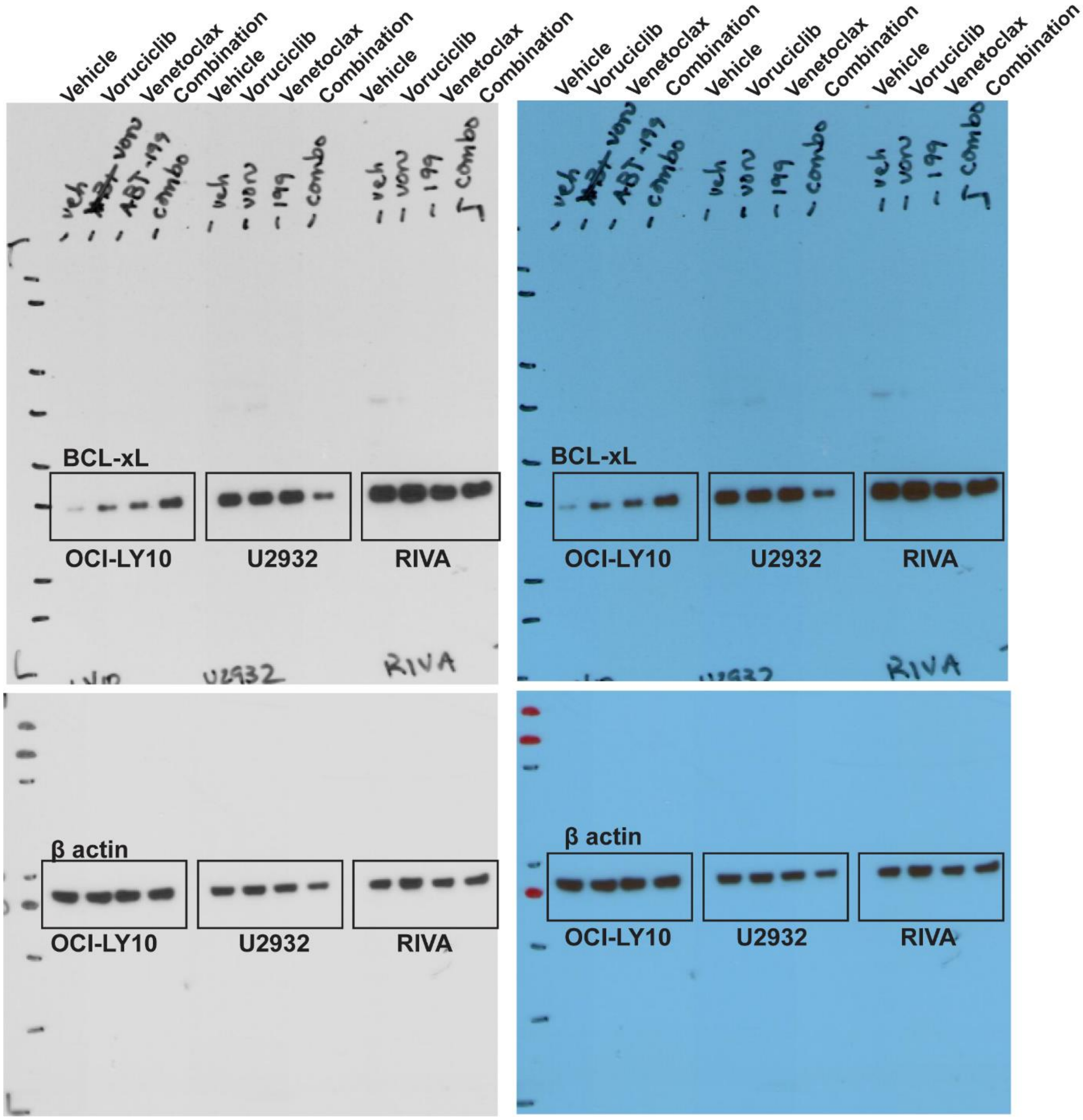

Supplement: Supplementary file 1 — Supplementary figures [file 41598_2017_18368_MOESM1_ESM.pdf]
